# HDACs control RUNX2 expression in cancer cells through redundant and cell context-dependent mechanisms

**DOI:** 10.1186/s13046-019-1350-5

**Published:** 2019-08-08

**Authors:** Gloria Manzotti, Federica Torricelli, Benedetta Donati, Valentina Sancisi, Mila Gugnoni, Alessia Ciarrocchi

**Affiliations:** Laboratory of Translational Research, Azienda Unità Sanitaria Locale – IRCCS di Reggio Emilia, Viale Risorgimento 80, 42123 Reggio Emilia, Italy

**Keywords:** RUNX2, HDACs, Gene expression regulation, HDAC inhibitors, Cancer

## Abstract

**Background:**

RUNX2 is a Runt-related transcription factor required during embryogenesis for skeletal development and morphogenesis of other organs including thyroid and breast gland. Consistent evidence indicates that RUNX2 expression is aberrantly reactivated in cancer and supports tumor progression. The mechanisms leading to RUNX2 expression in cancer has only recently began to emerge. Previously, we showed that suppressing the activity of the epigenetic regulators HDACs significantly represses RUNX2 expression highlighting a role for these enzymes in RUNX2 reactivation in cancer. However, the molecular mechanisms by which HDACs control RUNX2 are still largely unexplored. Here, to fill this gap, we investigated the role of different HDACs in RUNX2 expression regulation in breast and thyroid cancer, tumors that majorly rely on RUNX2 for their development and progression.

**Methods:**

Proliferation assays and evaluation of RUNX2 mRNA levels by qRT-PCR were used to evaluate the effect of several HDACi and specific siRNAs on a panel of cancer cell lines. Moreover, ChIP and co-IP assays were performed to elucidate the molecular mechanism underneath the RUNX2 transcriptional regulation. Finally, RNA-sequencing unveiled a new subset of genes whose transcription is regulated by the complex RUNX2-HDAC6.

**Results:**

In this study, we showed that Class I HDACs and in particular HDAC1 are required for RUNX2 efficient transcription in cancer. Furthermore, we found an additional and cell-specific function of HDAC6 in driving RUNX2 expression in thyroid cancer cells. In this model, HDAC6 likely stabilizes the assembly of the transcriptional complex, which includes HDAC1, on the RUNX2 P2 promoter potentiating its transcription. Since a functional interplay between RUNX2 and HDAC6 has been suggested, we used RNA-Seq profiling to consolidate this evidence in thyroid cancer and to extend the knowledge on this cooperation in a setting in which HDAC6 also controls RUNX2 expression.

**Conclusions:**

Overall, our data provide new insights into the molecular mechanisms controlling RUNX2 in cancer and consolidate the rationale for the use of HDACi as potential pharmacological strategy to counteract the pro-oncogenic program controlled by RUNX2 in cancer cells.

**Electronic supplementary material:**

The online version of this article (10.1186/s13046-019-1350-5) contains supplementary material, which is available to authorized users.

## Background

RUNX2 is a member of the mammalian RUNT related transcription factor family, necessary during embryogenesis for skeletal development [1–3] and for the morphogenesis of other organs like breast and thyroid [4, 5]. As many other factors crucial for embryogenesis, RUNX2 is often aberrantly reactivated in cancer. Indeed several studies reported the over-expression of RUNX2 in tumor derived from epithelial tissues, including: thyroid [6, 7], breast [8], pancreas [9, 10], prostate [11], lung [12, 13], melanoma [14], glioma [15], colorectal [16] and osteosarcoma [17]. The RUNX2 gene encodes two major isoforms starting from two alternative promoters [18, 19], the isoform I, controlled by the proximal P2 promoter, is the major RUNX2 isoform in tumor cells [6, 20, 21].

The regulatory mechanisms that control the activity of the P2 promoter and that lead to RUNX2 re-expression in cancer have been unknown for long time. Recently, we demonstrated that the P2 promoter has a limited transcription activity in different cancer models [20]. Moreover, we showed that RUNX2 expression is regulated by a network of non-redundant ENHs that cooperate with the P2 promoter through the selective binding of specific TFs and through chromatin topological conformation [22]. These ENHs are the final target of different pathways already known to affect RUNX2 expression such as FGFR-MAPK axis, TGFβ and BMP through SMAD proteins and c-JUN, member of the AP1 family of TFs.

The ability of RUNX2 to enhance the metastatic potential of tumor cells is largely based on its ability to regulate genes crucial to tumor progression including VEGF, MMP9, MMP13, OPN, SNAI 1–2, TWIST1 and TIMP13 [3, 6, 23–29]. The oncogenic role of RUNX2 is dependent on the cellular context and it is affected by cell specific post translational mechanisms and availability of transcription partners [6]. RUNX2 has been shown to interact with several Transcription Factors (TFs) and with many co-factors including different HDACs. The functional interplay between RUNX2 and HDACs is rather complicated. RUNX2 can be either partner and target of the activity of these enzymes and the overall effects on RUNX2 transcriptional function is different depending on the context.

Protein acetylation is a highly specific post-translational modification that largely affects gene expression by defining both accessibility of chromatin and activity of many non-histone proteins that directly or indirectly participate to transcription regulation. The overall acetylation program in the cells is defined by the coordinated activity of two classes of enzymes: Histone Acetyl Transferases (HATs) whose function is to add Acetyl group to target proteins and Histone DeAcetylases (HDACs) that revert HATs activity by removing Acetyl group from target proteins. Histones are major targets of these enzymes. Histone acetylation (in particular H3K27Ac and H3K9Ac) is associated with chromatin accessibility and gene expression activation. Loading histones with the negative charge of the acetyl group loosens the binding of histones with DNA, leading to a more open chromatin structure. HDAC superfamily consists of 11 components divided in four classes (I, IIa, IIb and IV) and seven sirtuins (referred to as class III). Most of these proteins are localized in the nucleus and classically considered transcriptional repressors, due to their histone de-acetylation activity. However, gene expression profiling and functional studies also highlighted the ability of these enzymes to directly enhance transcription by controlling the activation status of non-histone transcriptional regulatory proteins [30, 31]. Inhibitors of HDACs have been proposed as promising anticancer strategies. According to the most credited models, blocking the activity of these enzymes would increase chromatin hyper-acetylation at the level of onco-suppressors regulatory elements, leading to their re-expression. However, the use of these drugs as monotherapy revealed to be effective only in hematological malignancies while failed to produce significant benefits for patients with solid cancer [32]. Filling the gaps in our understanding of the HDACs mechanisms of action will likely help overcoming these limitations, insuring the appropriate use of these drugs in the clinical setting.

We recently reported that HDACi inhibits RUNX2 expression in several cancer types and that the strength of this inhibition is tightly dependent on RUNX2 levels of expression [20]. Our data also indicate that different HDACs are involved in supporting RUNX2 expression depending on the cell types even if the molecular mechanisms by which this regulation takes place are still largely unknown.

In this work we aimed to explore the way HDACs control the expression of RUNX2 in cancer cells pointing at clarifying which HDACs are involved and their mechanisms of action.

## Methods

### Cell cultures, treatments and proliferation assays

A375, BCPAP, TPC1, MDA-MB231 were cultured in DMEM, H1299 and PC3 were cultured in RPMI and HCT-116 were cultured in IMDM; all cell lines were grown at 37 °C*/*5% CO_2_ in medium added with 10% fetal bovine serum and 1% penicillin – streptomycin. All cell lines were routinely tested for Mycoplasma contamination and authenticated by SNP profiling at Multiplexion GmbH (Heidelberg, Germany), last authentication was performed in January 2019. All cell lines were treated for 24–48 – 72 h (depending on the assay performed) with different concentrations of the following drugs: Tubacin, SAHA, Valproic acid (Sigma-Aldrich, St. Louis, Missouri, USA), TMP-269, PCI-34051, 4SC-202 (Selleckchem, Munich, Germany) or the respective control. All drugs were resuspended in DMSO (Sigma-Aldrich, St. Louis, Missouri, USA) except for Valproic acid which were reconstituted in water. For proliferation assays, treated cells were counted by trypan blue exclusion with Countess® Automated Cell Counter (Thermo Scientific, Waltham, Massachusetts, USA).

### siRNA transfections

Cells were reverse-transfected with RNAiMax Lipofectamine (Thermo Scientific, Waltham, Massachusetts, USA) and harvested 48 h after transfection for further analysis. siRNA used were: HDAC1, HDAC2, HDAC3 and HDAC8 Silencer Select RNAi (Thermo Scientific, Waltham, Massachusetts, USA) at a final concentration of 30 nM; HDAC6 TriFECTa DsiRNA Duplex (Integrated DNA Technologies, Coralville, Iowa, USA) at a final concentration of 10 nM each duplex; RUNX2 Stealth RNAi (Thermo Scientific, Waltham, Massachusetts, USA) at a final concentration of 10 nM each oligo. For each type of siRNA, the corresponding negative control were used. For simultaneously silencing of HDAC1, HDAC2 and HDAC3, the final concentration of each specific oligos was 30 nM. See Additional file 2: Table S1 for oligos’ sequences and/or reference code.

### Quantitative real-time PCR

Total RNA was extracted from treated cells with Maxwell®RSC simplyRNA Cells (Promega, Madison, Wisconsin, USA) and retrotranscribed with iScript cDNA kit (Bio-Rad, Hercules, California, USA). Quantitative Real-Time PCR (qRT-PCR) was performed using GoTaq® qPCR Master Mix (Promega, Madison, Wisconsin, USA) in a CFX96 Real Time PCR Detection System (Bio-Rad, Hercules, California, USA). Relative expression of target genes was calculated using the ΔΔCt method by normalizing to the reference gene expression Beta-DGlucuronidase (GUSB). For RNA-Seq validation normalization was performed with the geometric mean of three reference genes expression: Hypoxanthine Phosphoribosyltransferase 1 (HPRT), Glyceraldehyde 3-phosphate dehydrogenase (GAPDH), Ribosomal Protein S17 (RPS17). See Additional file 2: Table S2 for qRT-PCR primers sequences.

### Chromatin immunoprecipitation

ChIP experiments were performed as previously described [22]. Briefly, after cross-linking with 1% formaldehyde, cells were lysed and chromatin sonicated with Bioruptor® Pico sonicator (Diagenode SA, Ougrée, Belgium) then precipitated with Magna ChIP™ Protein G Magnetic Beads (16–662, Millipore, Burlington, Massachusetts, USA) and the appropriate antibody (Additional file 2: Table S3). The immunoprecipitated DNA fragments were analyzed by qPCR, see Additional file 2: Table S1 for primers sequences. For each experiment, a chromatin amount corresponding to 1% of chromatin used for immunoprecipitation was kept as input control. Each qPCR value was normalized over the appropriate input control and reported in graphs as input %. (qPCR value*/*input value × 100).

### Co-immunoprecipitation and Western blot analysis

For co-immunoprecipitation experiments a fractionation of cytoplasmic and nuclear proteins cells were harvested and washed in PBS. A small aliquots were lysed with PLB (Promega, Madison, Wisconsin, USA) in order to obtain a total lysate; than cytoplasm lysis were performed by incubating on ice for 4 to 8 min (for MDA-MB231 and TPC1 respectively) on Cytosol Buffer (10 mM HEPES pH 7.9, 1,5 mM MgCl2, 10 mM KCl, 0,5% NP-40, 1X protease inhibitor). After lysis of the cytoplasm, nuclei were resuspended in Lysis Buffer (50 mM Tris-HCl pH 7,4, 150 mM NaCl, 1 mM EDTA, 1% Triton-X, 1X protease inhibitor) and incubated at + 4 °C for 30 min. Then, soluble proteins were separated from debris by 10 min centrifugation at 1200 rpm and quantified with Bradford Protein Assay (Bio-Rad, Hercules, California, USA). An equal amount of proteins was precipitated with Protein A Sepharose CL-4B (GE Healthcare, Chicago, Illinois, USA) and the appropriate antibody (Additional file 2: Table S3). Western blot analysis was performed as previously described [33] with antibodies listed in Additional file 2: Table S3.

### RNA-Seq and Bioinformatic analysis

RNA was quantified by Nanodrop (Thermo Scientific, Waltham, Massachusetts, USA) and quality assessment was performed by Bioanalyzer -RNA 6000 nano kit (Agilent Technologies, Santa Clara, California, USA).

Libraries were prepared starting from 1 μg RNA using TruSeq Stranded mRNA kit (Illumina, San Diego, California, USA). Next generation sequencing was conducted on NextSeq 500 platform (Illumina, San Diego, California, USA) and a minimum of 30 million of reads for each replicate was expected. Cufflink RNA-Seq workflow was applied to perform bioinformatic analysis. Differential gene expression was calculated as log2 fold-change (siRUNX2/siCtrl, siHDAC6/siCtrl). Differential expression *p*-values were adjusted by an optimized FDR approach (FDR cutoff = 0.05) and genes with adjusted *p*-value (q-value) < 0.05 were considered significantly deregulated.

### Statistical analysis

Statistical analysis was performed using GraphPad Prism Software (GraphPad Software, San Diego, California, USA). Statistical significance was determined using the Student’s *t*-test. R library “ggpubr” was used to perform correlation analysis between RUNX2 and HDAC6 expression in thyroid tumor tissues from 502 patients. Patient’s data were extracted from TCGA-THCA project using R library “TCGAbiolinks”.

## Results

### HDAC1 is required for efficient RUNX2 transcription

**Fig. 1:**
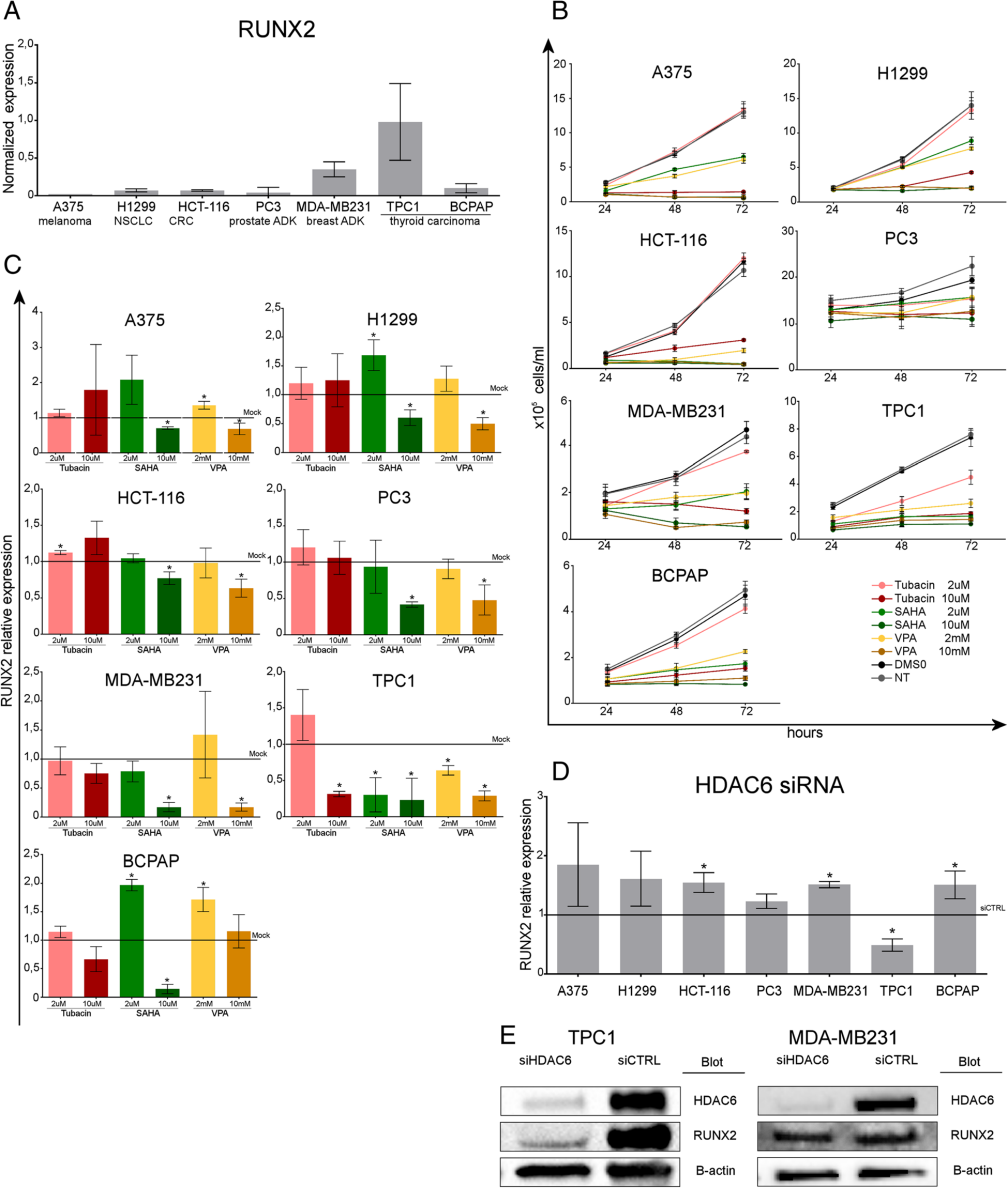
HDAC regulates RUNX2 expression in cancer. A375, H1299, HCT-116, PC3, MDA-MB231, TPC1 and BCPAP cancer cell lines were tested for their RUNX2 expression levels by qRT-PCR (**a**). All cells were treated with low and high doses of tubacin (specific HDAC6 inhibitor) and two different pan-HDACi (SAHA and valproic acid), then proliferation (**b**) and RUNX2 expression levels (**c**) were evaluated. All cell lines were treated with specific siRNA against HDAC6, 48 h after transfection RUNX2 levels were assessed by qRT-PCR in all cell lines (**d**) and by Western Blot in TPC1 and MDA-MB231 as a representative control (**e**). For proliferation experiments, cell counts were performed at 24–48-72 h after treatment, graphs show a representative experiment performed in triplicate. Histograms represents the mean relative fold change +/− SD of treated cells compared to control cells. Each experiment represents the average of at least two independent replicates. * *p* < 0.05

To analyze the contribution of HDACs to RUNX2 regulation and the effect of their inhibition in cancer biology, we selected a panel of cell lines derived from tumor types in which RUNX2 has been shown to be involved. qRT-PCR analysis confirms that thyroid and breast cancer cells express the highest RUNX2 levels among the testes cell lines (Fig. 1a).

Next, each cell line was treated with three different HDACi: SAHA which is a panHDACi, Tubacin that is a specific inhibitor for HDAC6 and Valproic acid (VPA) which specifically inhibits class I and IIa HDACs. This choice was based on our previous observation that in thyroid TPC1 cells HDAC6 was required for RUNX2 expression [20]. Thus, we also wanted to test whether this peculiar HDAC is involved in RUNX2 regulation also in other settings. Figure 1b shows the growth curves of these cell lines treated with two different concentrations of SAHA (2 uM, 10 uM), Tubacin (2 uM, 10 uM) and VPA (2 mM and 10 mM). With the exception of PC3, all cell lines showed a good and dose dependent sensitivity to SAHA and VPA. By contrast, tubacin was effective only at the highest concentration, with the exception of TPC1 which were highly sensitive to this drug even at the lowest dose.

**Fig. 2:**
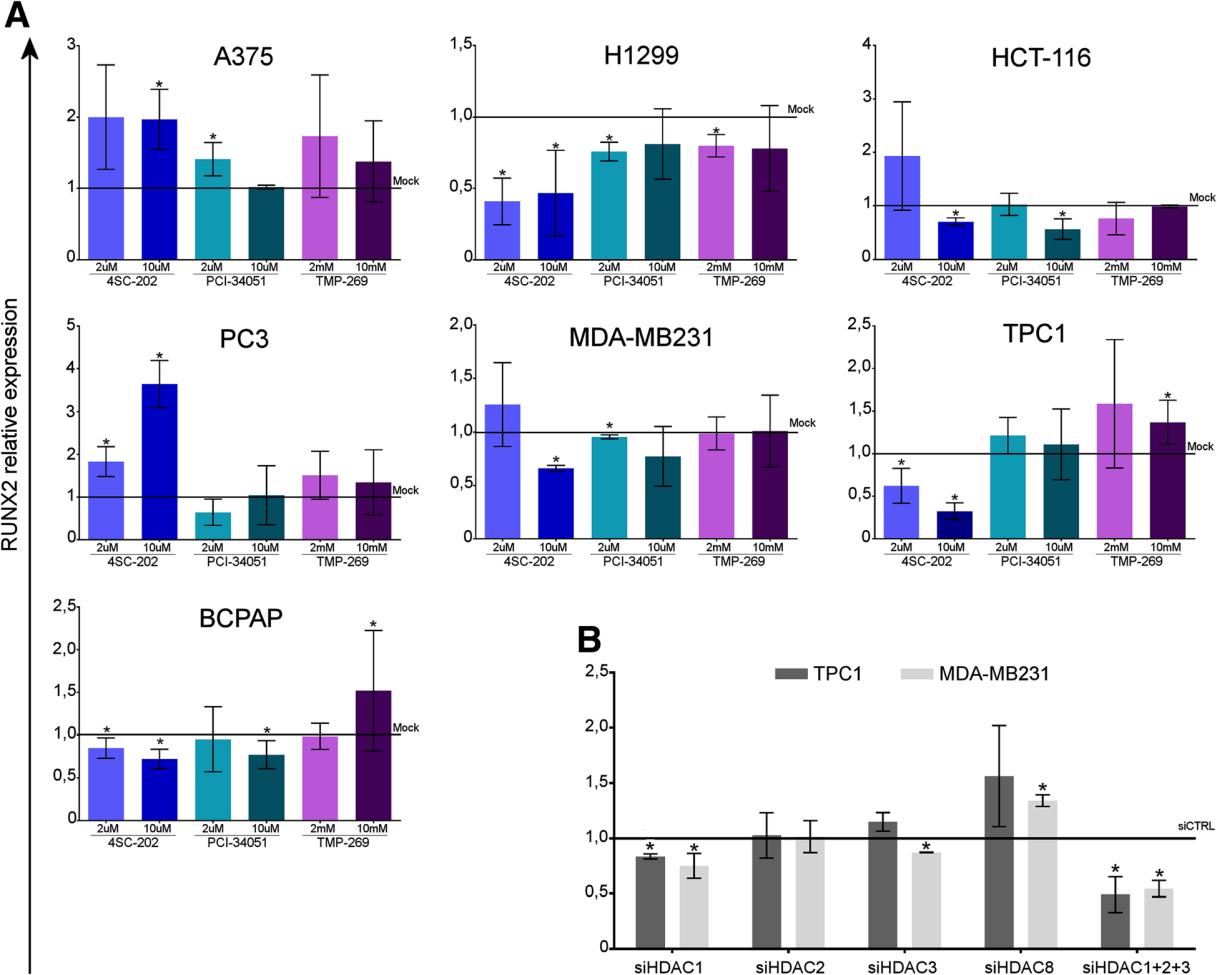
RUNX2 expression in cancer depends primarily on HDACs class I. All cell lines were treated for 48 h with low and high doses of HDACs class I specific inhibitors: 4SC-202 (domatinostat, specific for HDAC 1–2-3) PCI-3405 (specific for HDAC8) and TMP269 (specific for class IIa) then RUNX2 levels were assessed by qRT-PCR (**a**). TPC1 and MDA-MB231 were transfected with siRNA specific for HDAC1, HDAC2, HDAC3 and HDAC8 or with the combination of the former three (**b**). Histograms represent the mean relative fold change +/− SD of treated /silenced cells compared to the respective control cells. Each experiment represents the average of at least two independent replicates. * *p* < 0.05

Next, we investigated RUNX2 expression in these cells upon drug exposure (Fig. 1c). Low doses of all HDACi induced no changes in RUNX2 levels or a slight increase in only two cell lines. This result is in line with the known effect of HDAC inhibitors that is a general enhancement of transcription. Conversely, in all cell lines, high doses of the pan-HDACi SAHA and VPA resulted in a significant inhibition of RUNX2 expression, in accordance with the negative effect on the proliferation of these cells. Besides, tubacin treatment resulted in a significant inhibition of RUNX2 only in TPC1. Being tubacin a HDAC6 specific inhibitor, these data indicate that HDAC6 is selectively involved in RUNX2 transcription in this thyroid cancer cell line, highlighting a close connection between proliferation inhibition and RUNX2 down-regulation. To further prove this hypothesis, we used HDAC6 specific siRNAs to silence it in all tested cell lines (Fig. 1d-e, Additional file 1: Figure S1a). Indeed, silencing HDAC6 resulted in a significant RUNX2 inhibition only in TPC1, confirming the data obtained with the specific inhibitors. Next, we aimed at characterizing which HDACs may be responsible for RUNX2 expression regulation in the other cell models. First, we took advantage of class-specific inhibitors to restrict our observation. To this end 4SC-202 (domatinostat, specific for HDAC 1–2-3) PCI-3405 (specific for HDAC8) and TMP269 (specific for class IIa) were employed. Figure 2a shows the effect of these drugs on RUNX2 expression in each cell line. None of the class-specific inhibitors recapitulate entirely the effects of the panHDACi. This suggest redundancy in the activity of these enzymes in RUNX2 regulation. Nevertheless, in the majority of the tested cell lines domatinostat showed the strongest effect. These results indicate that class I HDACs are likely a relevant part of the transcriptional apparatus that support RUNX2 expression in cancer cells.

To further consolidate this evidence, we performed siRNA against HDAC class I members in TPC1 and MDA-MB231, the cell lines with the highest RUNX2 expression. Silencing HDAC1 resulted in a significant reduction of RUNX2 expression in both cell lines even if the strength of this effect was limited in TPC1, compared to the effect of HDAC6 knockdown. Silencing HDAC3 showed a mild effect only in MDA-MB231, while silencing HDAC2 had no effect on RUNX2 expression in both cell lines. In accordance with the results obtained with PCI-3405, no repression of RUNX2 expression was observed upon HDAC8 silencing. Simultaneous silencing of HDAC1,2 and 3 resulted in a consistent RUNX2 repression in both TPC1 and MDA-MB231 (Fig. 2b, Additional file 1: Figure S1c-d). Similar results were obtained in BCPAP, an additional cell model of thyroid cancer (Additional file 1: Figure S1b-d). Taken together, these experiments indicate that HDAC1 promotes RUNX2 expression in these cells. In its absence, HDAC2 and HDAC3 may partially vicariate its function. Furthermore, in TPC1, RUNX2 expression requires the additional and cell-specific cooperation of HDAC6.

### HDAC6 stabilizes transcriptional complex, promoting RUNX2 expression in TPC1 cells

TPC1 cells display significantly higher levels of RUNX2 compared to the rest of tested cancer cell lines, including MDA-MB231. We hypothesize that HDAC6 may be responsible for this difference, being this enzyme selectively required for RUNX2 expression in this cell line. To test this hypothesis, we explored the role of HDAC6 and HDAC1 in RUNX2 transcription in TPC1. We also tested their function in MDA-MB231 where HDAC6 does not participate to RUNX2 expression regulation. First, we investigated the binding of these proteins on the regulatory elements of RUNX2 gene. We previously showed that RUNX2 isoform I is the only expressed in cancer and that the RUNX2-P2 transcriptional activity in thyroid and breast cancer is regulated by its interaction with one proximal (ENH3) and two distal (ENH11 and ENH13) enhancers (ENHs) [20, 22].

**Fig. 3:**
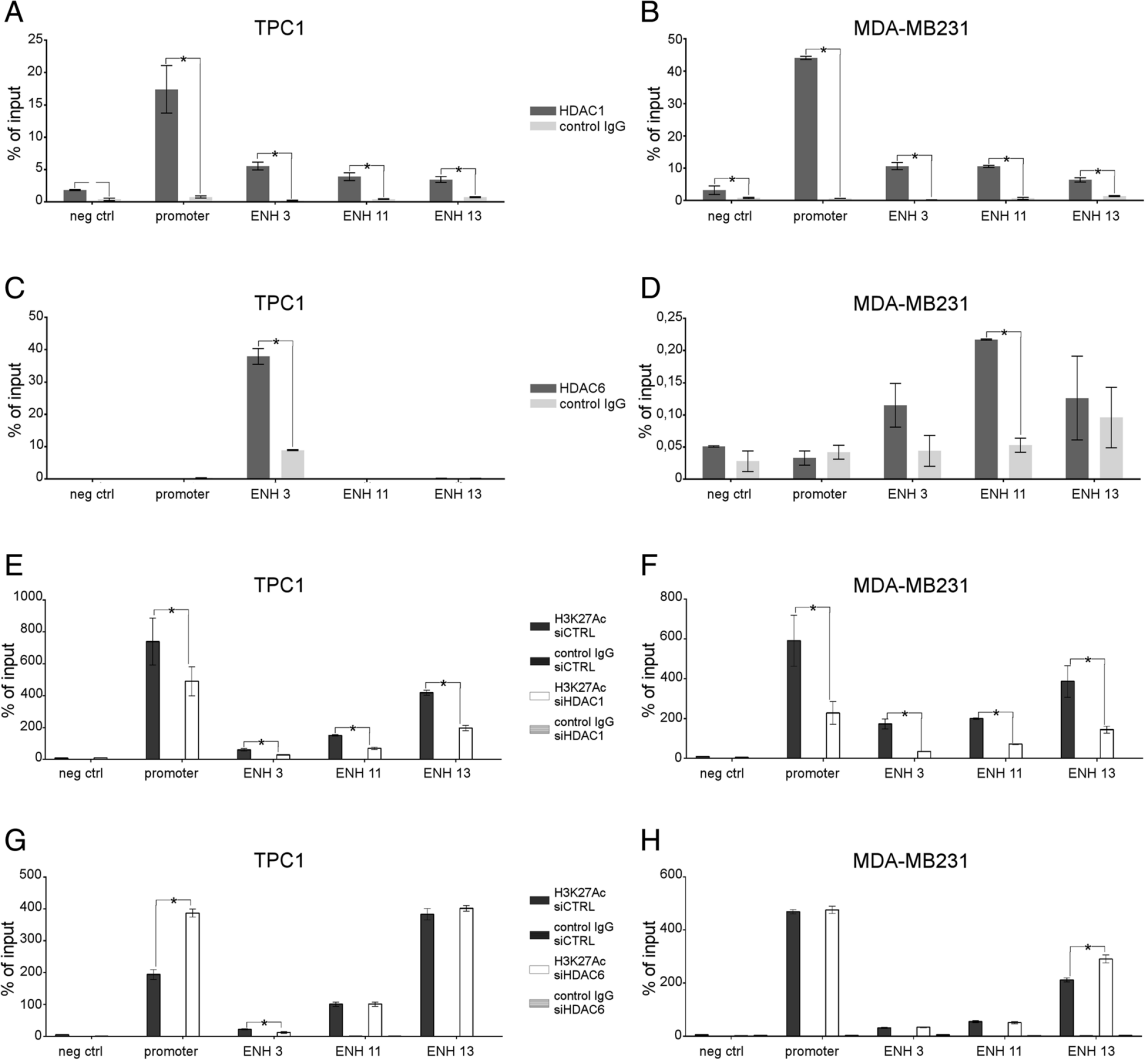
HDAC1 and HDAC6 bind RUNX2 regulatory elements and control their transcriptional activation status. TPC1 and MDA-MB-231 were assessed for the binding of HDAC1 (**a-b**) and HDAC6 (**c**-**d**) to the regulatory elements of RUNX2 by ChIP assays. ChIP experiments were also performed to evaluate levels of H3K27Ac in RUNX2 regulatory regions 48 h after transfection with siRNA specific for HDAC1 (**e**-**f**) or HDAC6 (**g**-**h**). Histograms represent the average enrichment of the indicated genomic regions in the immunoprecipitated DNA expressed as percentage of the Input. All data are expressed as mean values +/− SEM of a technical triplicate and are representative of at least two independent experiments. * *p* < 0.05

The results of chromatin immuno-precipitation (ChIP) with HDAC1 and HDAC6 antibodies on RUNX2 P2 promoter and ENHs are shown in Fig. 3a-d. In both cell lines, HDAC1 is strongly enriched on RUNX2 P2. A significant, but sensibly lower binding was also observed on ENH3, ENH11 and ENH13 in both models. Similar results were obtained in BCPAP cells (Additional file 1: Figure S1e). HDAC6 binding was strongly enriched on ENH3 in TPC1, while its presence on RUNX2 regulatory elements on MDA-MB231 is extremely faint and probably with no biological relevance. Notably, the nuclear amount of HDAC6 was similar in the two cell lines (Additional file 1: Figure S1f). These data are in agreement with the specific dependency of RUNX2 expression on HDAC6 in TPC1 cells. Furthermore, being HDAC6 primarily a cytoplasmic protein, these observations identify RUNX2 as one of the few direct transcriptional targets of this enzyme. Next, we investigated the effect of HDAC1 and HDAC6 on the transcriptional activity of RUNX2 regulatory elements. To this end, HDAC1 and HDAC6 were silenced in both TPC1 and MDA-MB231 and the enrichment of H3K27AC (marker of active transcription) on RUNX2 P2 promoter and ENHs were investigated by ChIP. Surprisingly, silencing of HDAC1 resulted in a partial but significant reduction of H3K27Ac levels at both promoter and ENHs in both cell lines (Fig. 3e-f). This observation, even if in apparent contrast with the expected deacetylation function of HDAC1, is in line with the effect of HDAC1 inhibition on RUNX2 expression.

In MDA-MB231 cells, HDAC6 silencing had no significant effect on H3K27AC distribution on RUNX2 gene. By contrast, in TPC1 cells, silencing HDAC6 increased H3K27Ac levels at the P2 promoter without affecting the acetylation status of the ENHs (Fig. 3g-h). Since HDAC6 does not directly bind to the RUNX2 P2 promoter, we speculated that this was a compensatory effect to overcome the transcriptional inhibition imposed by HDAC6 silencing.

**Fig. 4:**
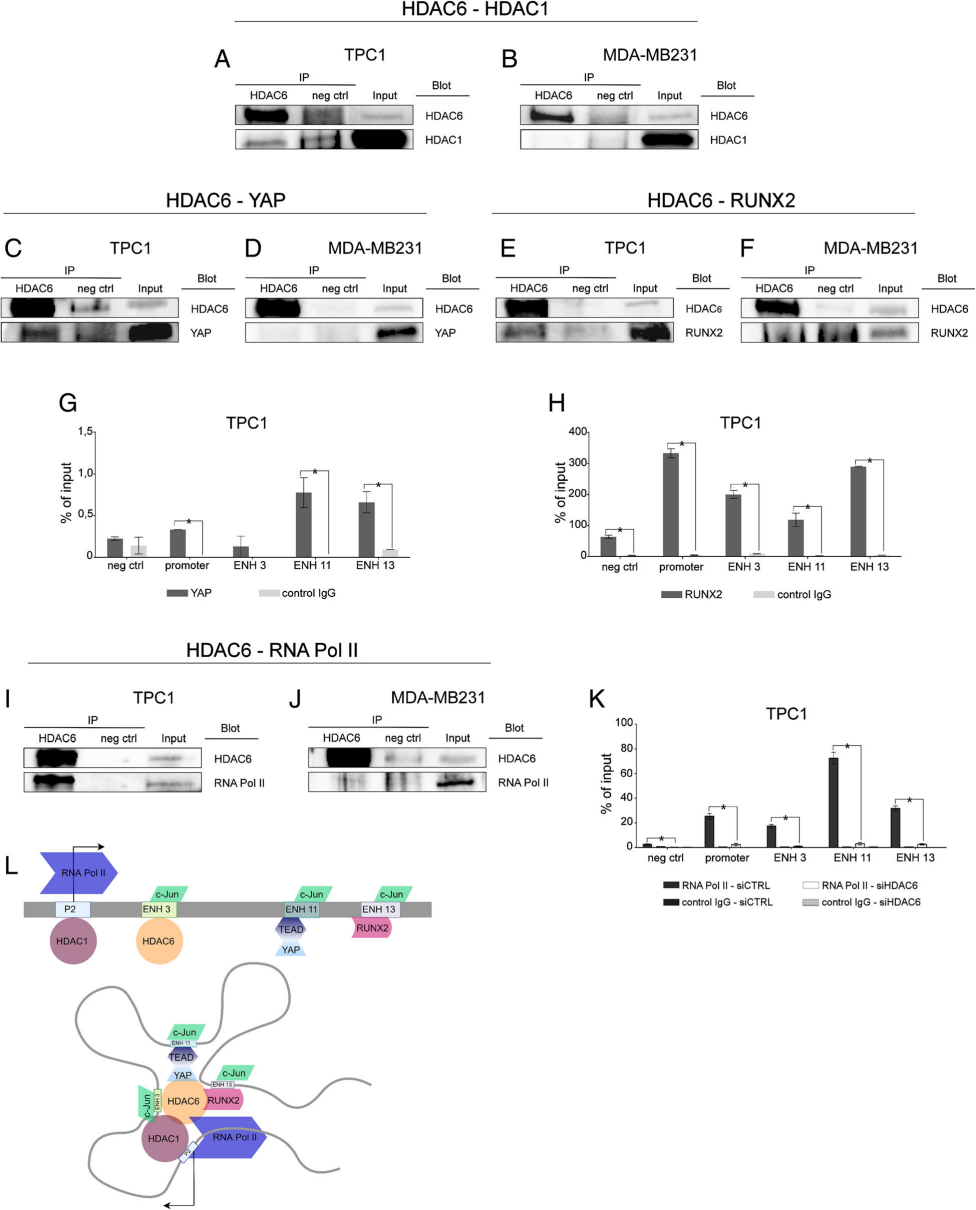
HDAC1 and HDAC6 cooperates to enhance RUNX2 expression in TPC1 thyroid cancer cells. Nuclear extract from TPC1 and MDA-MB231 cells were tested for the presence of a multi-protein complex controlling RUNX2 expression. Co-immunoprecipitation experiments were performed to evaluate binding of HDAC6 to HDAC1 (**a**-**b**), YAP (**c**-**d**), RUNX2 (**e**-**f**) and RNA Pol II (**i**-**j**). Western blots are representative of two independent experiments. ChIP assay show binding of YAP (**g**) and RUNX2 (**h**) to RUNX2 regulatory elements. ChIP experiments were also performed to evaluate levels of RNA Pol II on RUNX2 regulatory elements 48 h after transfection with siRNA specific for HDAC6 (**k**). Histogram represent the average enrichment of the indicated genomic regions in the immunoprecipitated DNA expressed as percentage of the Input. Data are expressed as mean values +/− SEM of a technical triplicate and are representative of at least two independent experiments. * *p* < 0.05. A schematic model illustrating how HDAC6 acts on RUNX2 transcription by stabilizing the interaction between the different regulating factors, thus enhancing the activity of the transcriptional complex (**l**)

These observations support the hypothesis that HDAC1 and HDAC6 cooperates to promote RUNX2 expression in TPC1 but not in MDA-MB231. Next, we investigated whether HDAC1 and HDAC6 could be part of the same transcriptional complex. Thus, we performed co-immunoprecipitation (co-IP) experiments in TPC1 and MDA-MB231 using HDAC6 antibody. Noticeably, HDAC1 co-immunoprecipitated with HDAC6 only in TPC1 (Fig. 4a) while no sign of interaction was detected in MDA-MB231 (Fig. 4b). Reverse immunoprecipitation with HDAC1 confirmed the interaction with HDAC6 only in TPC1, consolidating these results (Additional file 1: Figure S1 g-h). In a recent work we identified c-JUN as master regulator of a transcriptional network that converge on RUNX2 ENHs, controlling its expression. We also showed that c-JUN binds to each of the three RUNX2 ENHs together with different transcriptional partners including YAP and RUNX2 itself, in a positive feedback loop [22]. Consistently with this model, co-IP with HDAC1 antibodies showed that c-JUN selectively interacts with the HDAC1- HDAC6 complex only in TPC1 but not in MDA-MB231 (Additional file 1: Figure S1 g-h). Moreover, co-IP with HDAC6 antibody indicate that both YAP and RUNX2 selectively interacted with HDAC6 only in TPC1 cells (Fig. 4c-f). Finally, ChIP experiments with YAP and RUNX2 antibodies confirm the enrichment of these TFs in the RUNX2 regulatory elements supporting the proposed model (Fig. 4g-h). To further sustain the selective requirement of HDAC6 for RUNX2 transcription in TPC1, we investigated its interaction with RNA-PolII. Noticeably HDAC6 co-immunoprecipitated with RNA-PolII only in TPC1 and not in MDA-MB231 cells (Fig. 4i-j). Indeed, silencing of HDAC6 in TPC1 determined a dramatic drop of RNA-PolII recruitment on RUNX2 P2 promoter and active ENHs (Fig. 4k). Taken together these data indicate that HDAC1, binding to the RUNX2 P2 promoter, prompts RUNX2 expression in cancer cells. In TPC1 cells, that heavily rely on RUNX2 expression, HDAC6 binds to ENH3 and stabilizes the tridimensional interaction of the transcriptional complex that drives RUNX2 expression, resulting in a further increase of its transcription (Fig. 4l). To extend these observations to human samples, we interrogated the TCGA data-set available for TC to explore the potential correlation between HDAC6 and RUNX2 in this context (*n* = 502). Noticeably a positive and significant correlation between RUNX2 and HDAC6 expression was observed consolidating our results (Additional file 1: Figure S1i).

### Transcriptional cooperation between RUNX2 and HDAC6 in thyroid cancer

Our data demonstrated that HDAC6 interacts with RUNX2 in TPC1 cells (Fig. 4c). Noticeably, recent reports have proposed a transcriptional cooperation between HDAC6 and RUNX2 in inhibiting p53-mediated apoptosis in cancer cells [34]. Thus, we explored whether HDAC6 assists RUNX2 in the regulation of specific target genes in thyroid cancer, beside their cooperation in controlling RUNX2 expression.

**Fig. 5:**
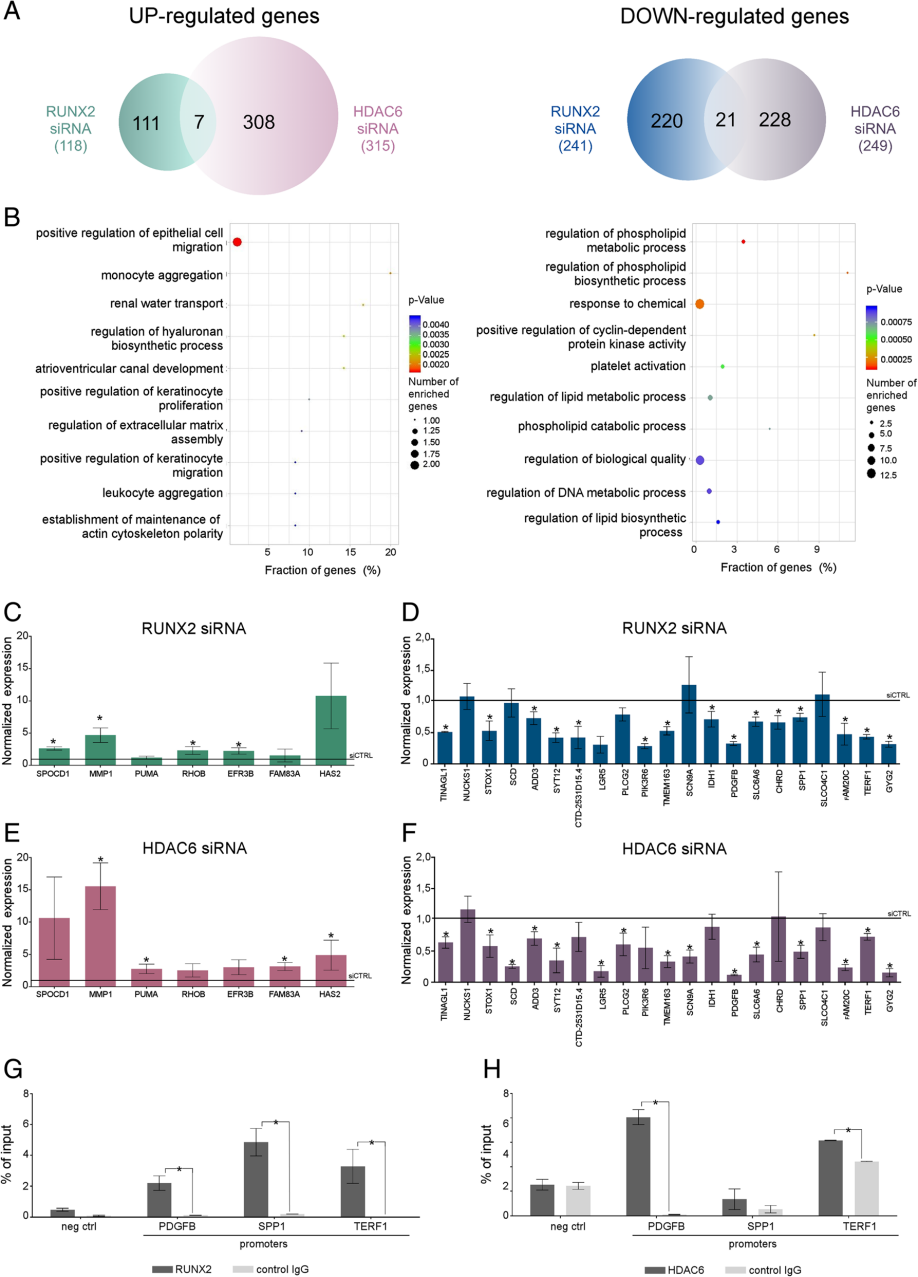
HDAC6 – RUNX2 complex controls a specific subset of target genes. TPC1 cells were transfected with siRNA against HDAC6 or RUNX2, after 48 h RNA-Seq analysis was performed. Venn diagram (**a**) and GO analysis (**b**) were used to analyze results. Common up- and down- regulated genes were validated on an independent set of RNAs (**c**-**f**). Histograms represent the relative fold change +/− SD of silenced cells as compared to control cells. Each experiment represents the average of three independent replicates. * *p* < 0.05. TPC1 were also assessed for the binding of RUNX2 (**g**) and HDAC6 (**h**) to the promoters of 3 of the genes regulated by the HDAC6-RUNX2 complex. Histograms represent the average enrichment of the indicated genomic regions in the immunoprecipitated DNA expressed as percentage of the Input. Data are expressed as mean values +/− SEM of a technical triplicate and are representative of at least two independent experiments. * *p* < 0.05

In order to identify the genes controlled simultaneously by RUNX2 and HDAC6, we performed RNA-Sequencing (RNA-Seq) in TPC1 cells upon silencing of RUNX2 or HDAC6 (Fig. 1e, Additional file 1: Figure S1j). Down-regulation of HDAC6 resulted in the deregulated expression of 564 genes of which 315 were up-regulated and 249 were down-regulated. Silencing of RUNX2 lead to alteration of 359 genes of which 118 were up-regulated and 241 down-regulated (Fig. 5a). Merge of significantly deregulated genes in these analyses identified a list of 28 genes that were coherently altered upon silencing of RUNX2 and HDAC6 indicating a possible cooperation of these two proteins in their regulation. 25% of common targets (7 of 28) were coherently induced by silencing of both proteins while the majority (75%, 21 of 28) were repressed when both RUNX2 and HDAC6 were silenced. These results suggest that, although RUNX2 and HDAC6 does not have many common targets, the main effect of this protein complex is transcriptional activation.

Up-regulated genes were enriched in migration and matrix interaction pathways. By contrast, gene ontology enrichment analysis on co-repressed genes identified predominantly metabolism related pathways (Fig. 5b). The matrix interacting protein SPP1, which is a consolidated RUNX2 target [35–37], was found among the genes that require RUNX2 and HDAC6 cooperation, while the remaining genes in this list have not been associated to RUNX2 transcriptional activity before. In order to validate these data, we tested a separate set of samples confirming the majority of the target genes identified by RNA-Seq analysis (Fig. 5c-f). To further explore the cooperation of RUNX2 and HDAC6 in inducing transcription of cancer related genes, we investigated their binding on the promoters of a selected set of genes identified as commonly repressed upon RUNX2 and HDAC6 silencing. To this purpose, we choose 3 of the most affected genes: SPP1 which is a well-known RUNX2 target; TERF1 a component of the telomere nucleoprotein complex which has been associated with poor outcome in different types of cancer [38, 39]; PDGFβ a sub-unit of the PDGF-receptor ligand and a potent mitogen for cells of mesenchymal origin, recently implicated in tumor-microenvironment interactions in several tumor settings [40, 41]; ChIP analysis in TPC1 cells showed that RUNX2 is significantly enriched on all tested promoters and in particular, on SPP1 promoter which is a consolidated RUNX2 target. HDAC6 binding was also observed on TERF1 and PDGFβ promoters but not on SPP1 promoter, suggesting that the transcriptional effect of HDAC6 silencing on this gene is likely caused by an indirect activity of this enzyme.

## Discussion

Many transcription factors that govern tissues and organs morphogenesis are hijacked during cancer progression. RUNX2 is not an exception to this paradigm but the mechanisms leading to RUNX2 expression in cancer remained unclear for long time. While RUNX2 isoform 2 expression is restricted to bone lineage, isoform 1, transcribed from the proximal P2 promoter, is the only RUNX2 variant expressed in cancer cells. Despite the low transcriptional activity of its promoter, RUNX2 isoform 1 expression levels are significant in the majority of cancer cells. To clarify this discrepancy, we recently identified three intergenic ENHs (ENH3, ENH11 and ENH13) that, brought together by chromatin tridimensional looping, cooperate to enhance and regulate RUNX2 transcription in cancer. The regulation by multiple ENHs helps ensuring the precision of expression patterning and contributes to phenotypic robustness. ENHs act as the keystone of many regulatory circuits and we showed that a precise set of extracellular signals converges on each of the identified RUNX2 ENHs, contributing to integrate and elaborate the received external information and modulating the expression of this TF. Each of these ENHs is bound by a precise set of TFs and we showed that c-JUN, binding on each of these elements is the nucleation center of this large transcriptional complex (Fig. 4l). Here, we report additional information about the assembly and mechanism of action of this complex showing that proficient RUNX2 expression in cancer cells requires the activity of HDACs. We previously reported that treatment with HDACi profoundly impairs RUNX2 expression in thyroid cancer. Here we brought further these observations by showing that HDAC1 binding at the RUNX2 P2 promoter is required for RUNX2 expression and that in cellular context with high level of RUNX2, such as TPC1 cells, HDAC6 promotes transcription by binding ENH3 and stabilizing the interplay between promoter and ENHs.

HDACs are central nodes in maintaining chromatin organization and functional flexibility. Being histone acetylation associated with increased transcriptional activity, HDACs have been historically associated with gene inactivation. In cancer, administration of HDACi is used to release chromatin silencing of tumor suppressors, leading to reintegration of their inhibitory function and consequential growth inhibition. For many years, this has been considered the main mechanism of action of these drugs as anticancer therapies [42, 43]. However, genome-wide evaluation of HDACi effects demonstrated that these drugs induce expression changes in 2 to 10% of all human genes with almost an equal amount of induced and repressed genes [44, 45]. Furthermore, the analysis of HDACs distribution across the genome, revealed the consistent accumulation of HDACs on active genes in agreement with a more articulated function of these enzymes on gene expression. In line with this non-canonical function, here we reported that HDACs are necessary to sustain the expression of RUNX2 in cancer cells and that treatment with HDACi inhibits this TF leading to a concomitant growth inhibition. Using class-specific inhibitors and selected siRNAs, we demonstrated that HDAC class I and in particular HDAC1 is largely responsible for this effect across different types of cancer. Based on our data, HDAC1 binds to the RUNX2 P2 promoter and only mildly to proximal and distal RUNX2 ENHs. Consistently, genome-wide analysis of HDAC1 distribution showed how this enzyme accumulates preferentially on DNAse hypersensitive sites within promoter regions of active genes. Intriguingly, at these sites, HDAC1 levels have been shown to correlate with RNA-PolII binding and high histone acetylation levels, leading to the hypothesis that this enzyme, in the context of active promoters, may facilitate transcription initiation. Indeed, consistently with this model, we observed that inhibition of HDAC1 alters the organization of the RUNX2 P2 promoter drastically reducing the H3K27Ac levels. Recently, it has been suggested that the activity of HDACs at active promoters serves to ensure efficient transcription by restraining promiscuous initiation [46], which would be consistent with the inhibitory effects observed on RUNX2 transcription upon HDACs inhibition. In addition to this mechanism, which is transversal to different types of cancers, we also showed that cancer cells characterized by peculiarly high RUNX2 levels (TPC1) rely on the additional activity of HDAC6.

Differently from the rest of HDACs, HDAC6 is mainly localized in the cytoplasm [47] where it deacetylates several substrates, such as Hsp90 [48], cortactin [49, 50] and tubulin, whose deacetylation affects microtubule (MT)-mediated processes [51–53]. For this reason, for long time the transcriptional function of this enzyme has been underestimated. HDAC6 is instead capable of shuttling between nucleus and cytoplasm due to the presence of a nuclear import/export signals (NLS/NES) [50]. Nuclear localization of HDAC6 seems to be triggered in response to specific stimuli, including cell cycle arrest and starvation [50, 54, 55], even if the number of known transcriptional targets of this HDAC is still limited. Similarly to other HDACs, genome-wide analysis of HDAC6 distribution, showed that this enzyme is recruited specifically at active genes (promoter and gene body) [46]. However, differently from HDAC1, HDAC6 seems to have a prevalent localization at the level of active ENHs. In line with this evidence, we showed that in TPC1, HDAC6 specifically binds ENH3, cooperating to the stabilization of the transcriptional complex required to drive RUNX2 expression. Indeed, we demonstrated that HDAC6 binds to c-JUN, YAP and RUNX2 selectively in TPC1 and that only in this context HDAC1 is detectable as part of this transcriptional complex. This suggests that the presence of HDAC6 facilitates the tridimensional interaction of these proteins and the communication of the TFs bound at the ENHs with the RUNX2 P2 promoter, enforcing RUNX2 transcriptional initiation. In line with this observation, we previously reported that treatment with HDACi in thyroid cancer cell lines causes the disassembly of the transcriptional complex bound at ENH3 [20]. Furthermore, here we showed that inhibition of HDAC6 in TPC1 leads to a dramatic drop of RNA-PolII recruitment at the RUNX2 P2 promoter and to the consequent inhibition of RUNX2 transcription. Recently, it has been hypothesized that HDAC6 is targeted to active genes through the direct interaction with elongating RNA-PolII [46]. This does not seem the case for RUNX2 expression, since, based on our data, the presence of HDAC6 on ENH3 is required for the binding of RNA-PolII on the RUNX2 P2 promoter.

Context-specific effects of HDACs on target genes have been largely reported (revised in [32]). However, in many settings, different HDACs may play redundant function. The regulation of RUNX2 expression in TPC1 offers an interesting example of how complex may be the functional relationship within the HDAC family. Indeed, here we report a highly specific context-dependent cooperation between HDAC1 and HDAC6 in which these two members of the HDAC family converge on the same target with interdependent but non-redundant function. Furthermore, while the function of HDAC1 may be compensated by other members of the Class I, loss of function of HDAC6 cannot be similarly overcome. Among the different cancer cell lines tested, this double activity of HDACs on RUNX2 seemed specific for TPC1. This cell line displays higher RUNX2 expression levels than the rest of the cell models evaluated in this study (Fig. 1a), underpinning the need of a more efficient transcription of this TF. On the other hand, there are no significant differences in the expression levels of HDAC6 (Additional file 1: Figure S1 j-k). The reason of this peculiarity remains to be elucidated.

Beside controlling its expression, HDACs have been shown to largely affect also RUNX2 transcriptional activity. In some cases, the interaction between RUNX2 and HDACs serves to mediate gene repression. HDAC6 has also been reported to interact with RUNX2 and affects its transcriptional activity [34]. In particular, it has been shown that HDAC6 interacts with RUNX2 and is recruited to RUNX2 target promoters where enhances the repressor activity of RUNX2 on pro-apoptotic genes, including p21.

To further elucidate the functional interplay between RUNX2 and HDAC6, we sought to investigate the transcriptional program of the RUNX2- HDAC6 complex, in thyroid cancer cells. With surprise, we noticed that the majority of common target genes were repressed upon HDAC6 or RUNX2 silencing, indicating the coordinated HDAC6-RUNX2 activity was associated with transcription activation rather than repression. Among the identified HDAC6-RUNX2 target genes we found SPP1, a consolidated RUNX2 target involved in cancer cells migration and invasiveness. However, the majority of identified genes were not previously associated to RUNX2 activity in cancer, providing new details about the transcriptional program sustained by this TF in cancer. Among these, we found mediators of oncogenic signals including WNT- pathway (LGR5), PDGF-pathway (PDGFβ) PI3K/AKT pathway (STOX1, PIK3R6). These observations reinforce the hypothesis that HDAC6 and RUNX2 cooperation serves for cancer progression. However, further experiments are required to elucidate the mechanisms by which HDAC6 helps RUNX2 in activating transcription, as well as to define the transversal meaning of their targets in other settings.

## Conclusions

In summary, our study adds new information about the complex function of HDACs in controlling gene expression, highlights context-specific cooperation among different members of the HDAC family. In particular, we provided additional details on the molecular mechanisms that drive RUNX2 aberrant expression in cancer and strengthen the rationale for the use of HDACi as potential therapeutic strategy to counteract the oncogenic program driven by RUNX2.

## Additional files

Additional file 1: **Figure S1.** a) Levels of HDAC6 mRNA in all cell lines 48 h after transfection with specific siRNA against HDAC6. b) RUNX2 expression levels 48 h after transfection with siRNA specific for HDAC1, HDAC2, HDAC3 and HDAC8 or with the combination of the former three, in BCPAP cells. c) expression levels of HDAC1, HDAC2, HDAC3 and HDAC8 48 h after transfection with the respective siRNA in TPC1, MDA-MB231 and BCPAP cells. d) expression levels of HDAC1, HDAC2 and HDAC3 48 h after transfection with a combination of the respective siRNAs in TPC1, MDA-MB231 and BCPAP cells. e) Representative ChIP experiments showing the binding of HDAC1 on the RUNX2 regulatory elements in BCPAP cells. f) Representative western blot showing basal levels of RUNX2, HDAC1 and HDAC6 in TPC1 and MDA-MB231 cells. C-Jun and ɑ-Tubulin were used as a control of correct nucleus-cytoplasm fractionation, this image is representative of all the fractionation performed for the co-IP experiments showed in Fig. 4g-h) Representative co-IP of HDAC1 with HDAC1 and c-Jun in TPC1 and MDA-MB231. i) Correlation of RUNX2 and HDAC6 expression in thyroid cancer samples from TCGA database. j) Representative western blot showing down-regulation of RUNX2 proteins 48 h after TPC1 cell transfection with specific siRNA. k) Basal expression levels of HDAC6 evaluated by qRT-PCR in all the tested cell lines. l) Correlation between the expression of RUNX2 and HDAC6. Where no otherwise specified, histograms represent the relative fold change +/− SD of silenced cells compared to control cells. Each experiment represents the average of at least two independent replicates. * *p* < 0.05 (TIF 3014 kb)

Additional file 2: Table S1-S3. List of Interfering oligos, Primers and Antobodies. (DOCX 28 kb)

## Data Availability

The datasets used and analyzed in the current study are available from the corresponding author on reasonable request.

## Abbreviations

ChIP: Chromatin immuno-precipitation
co-IP: Co-immunoprecipitation
ENH: Enhancer
GAPDH: Glyceraldehyde 3-phosphate dehydrogenase
GUSB: Beta-Dglucuronidase
HAT: Histone acetyl transferase
HDAC: Histone deacetylase
HDACi: HDAC inhibitor
HPRT: Hypoxanthine phosphoribosyltransferase 1
qRT-PCR: Quantitative Real-Time PCR
RNA-Seq: RNA sequencing
RSP17: Ribosomal protein S17
TF: Transcription factor
VPA: Valproic acid

## References

[1] Komori T, Yagi H, Nomura S, Yamaguchi A, Sasaki K, Deguchi K (1997). Targeted disruption of Cbfa1 results in a complete lack of bone formation owing to maturational arrest of osteoblasts. Cell.

[2] Lee B, Thirunavukkarasu K, Zhou L, Pastore L, Baldini A, Hecht J (1997). Missense mutations abolishing DNA binding of the osteoblast-specific transcription factor OSF2/CBFA1 in cleidocranial dysplasia. Nat Genet.

[3] Komori T (2018). Runx2, an inducer of osteoblast and chondrocyte differentiation. Histochem Cell Biol.

[4] Ferrari N, McDonald L, Morris JS, Cameron ER, Blyth K (2013). RUNX2 in mammary gland development and breast cancer. J Cell Physiol.

[5] Endo T, Kobayashi T (2010). Runx2 deficiency in mice causes decreased thyroglobulin expression and hypothyroidism. Mol Endocrinol.

[6] Sancisi V, Borettini G, Maramotti S, Ragazzi M, Tamagnini I, Nicoli D (2012). Runx2 isoform I controls a panel of proinvasive genes driving aggressiveness of papillary thyroid carcinomas. J Clin Endocrinol Metab.

[7] Han M, Chen L, Wang Y (2018). Overexpression suppresses tumorigenesis of papillary thyroid cancer via inactivation of PTEN/PI3K/AKT pathway by targeting Runx2. Onco Targets Ther.

[8] Chang CH, Fan TC, Yu JC, Liao GS, Lin YC, Shih AC (2014). The prognostic significance of RUNX2 and miR-10a/10b and their inter-relationship in breast cancer. J Transl Med.

[9] Kayed H, Jiang X, Keleg S, Jesnowski R, Giese T, Berger MR (2007). Regulation and functional role of the Runt-related transcription factor-2 in pancreatic cancer. Br J Cancer.

[10] Ozaki T, Yu M, Yin D, Sun D, Zhu Y, Bu Y (2018). Impact of RUNX2 on drug-resistant human pancreatic cancer cells with p53 mutations. BMC Cancer.

[11] Lim M, Zhong C, Yang S, Bell AM, Cohen MB, Roy-Burman P (2010). Runx2 regulates survivin expression in prostate cancer cells. Lab Investig.

[12] Li H, Zhou RJ, Zhang GQ, Xu JP (2013). Clinical significance of RUNX2 expression in patients with nonsmall cell lung cancer: a 5-year follow-up study. Tumour Biol.

[13] Herreño AM, Ramírez AC, Chaparro VP, Fernandez MJ, Cañas A, Morantes CF (2019). Role of RUNX2 transcription factor in epithelial mesenchymal transition in non-small cell lung cancer lung cancer: epigenetic control of the RUNX2 P1 promoter. Tumour Biol.

[14] Valenti MT, Dalle Carbonare L, Mottes M (2018). Ectopic expression of the osteogenic master gene. World J Stem Cells.

[15] Yamada D, Fujikawa K, Kawabe K, Furuta T, Nakada M, Takarada T (2018). RUNX2 promotes malignant progression in glioma. Neurochem Res.

[16] Ji Q, Cai G, Liu X, Zhang Y, Wang Y, Zhou L (2019). MALAT1 regulates the transcriptional and translational levels of proto-oncogene RUNX2 in colorectal cancer metastasis. Cell Death Dis.

[17] Kurek KC, Del Mare S, Salah Z, Abdeen S, Sadiq H, Lee SH (2010). Frequent attenuation of the WWOX tumor suppressor in osteosarcoma is associated with increased tumorigenicity and aberrant RUNX2 expression. Cancer Res.

[18] Banerjee C, Javed A, Choi JY, Green J, Rosen V, van Wijnen AJ (2001). Differential regulation of the two principal Runx2/Cbfa1 n-terminal isoforms in response to bone morphogenetic protein-2 during development of the osteoblast phenotype. Endocrinology.

[19] Li YL, Xiao ZS (2007). Advances in Runx2 regulation and its isoforms. Med Hypotheses.

[20] Sancisi V, Gandolfi G, Ambrosetti DC, Ciarrocchi A (2015). Histone deacetylase inhibitors repress Tumoral expression of the Proinvasive factor RUNX2. Cancer Res.

[21] Kammerer M, Gutzwiller S, Stauffer D, Delhon I, Seltenmeyer Y, Fournier B (2013). Estrogen receptor α (ERα) and estrogen related receptor α (ERRα) are both transcriptional regulators of the Runx2-I isoform. Mol Cell Endocrinol.

[22] Sancisi V, Manzotti G, Gugnoni M, Rossi T, Gandolfi G, Gobbi G (2017). RUNX2 expression in thyroid and breast cancer requires the cooperation of three non-redundant enhancers under the control of BRD4 and c-JUN. Nucleic Acids Res.

[23] Pratap J, Javed A, Languino LR, van Wijnen AJ, Stein JL, Stein GS (2005). The Runx2 osteogenic transcription factor regulates matrix metalloproteinase 9 in bone metastatic cancer cells and controls cell invasion. Mol Cell Biol.

[24] Selvamurugan N, Partridge NC (2000). Constitutive expression and regulation of collagenase-3 in human breast cancer cells. Mol Cell Biol Res Commun.

[25] Zelzer E, Glotzer DJ, Hartmann C, Thomas D, Fukai N, Soker S (2001). Tissue specific regulation of VEGF expression during bone development requires Cbfa1/Runx2. Mech Dev.

[26] Inman CK, Shore P (2003). The osteoblast transcription factor Runx2 is expressed in mammary epithelial cells and mediates osteopontin expression. J Biol Chem.

[27] Wang X, Manner PA, Horner A, Shum L, Tuan RS, Nuckolls GH (2004). Regulation of MMP-13 expression by RUNX2 and FGF2 in osteoarthritic cartilage. Osteoarthr Cartil.

[28] Niu DF, Kondo T, Nakazawa T, Oishi N, Kawasaki T, Mochizuki K (2012). Transcription factor Runx2 is a regulator of epithelial-mesenchymal transition and invasion in thyroid carcinomas. Lab Investig.

[29] Sun X, Wei L, Chen Q, Terek RM (2009). HDAC4 represses vascular endothelial growth factor expression in chondrosarcoma by modulating RUNX2 activity. J Biol Chem.

[30] Schroeder TM, Kahler RA, Li X, Westendorf JJ (2004). Histone deacetylase 3 interacts with runx2 to repress the osteocalcin promoter and regulate osteoblast differentiation. J Biol Chem.

[31] Westendorf JJ, Zaidi SK, Cascino JE, Kahler R, van Wijnen AJ, Lian JB (2002). Runx2 (Cbfa1, AML-3) interacts with histone deacetylase 6 and represses the p21(CIP1/WAF1) promoter. Mol Cell Biol.

[32] Manzotti Gloria, Ciarrocchi Alessia, Sancisi Valentina (2019). Inhibition of BET Proteins and Histone Deacetylase (HDACs): Crossing Roads in Cancer Therapy. Cancers.

[33] Gugnoni M, Sancisi V, Gandolfi G, Manzotti G, Ragazzi M, Giordano D (2017). Cadherin-6 promotes EMT and cancer metastasis by restraining autophagy. Oncogene.

[34] Ozaki T, Wu D, Sugimoto H, Nagase H, Nakagawara A (2013). Runt-related transcription factor 2 (RUNX2) inhibits p53-dependent apoptosis through the collaboration with HDAC6 in response to DNA damage. Cell Death Dis.

[35] Wai PY, Mi Z, Gao C, Guo H, Marroquin C, Kuo PC (2006). Ets-1 and runx2 regulate transcription of a metastatic gene, osteopontin, in murine colorectal cancer cells. J Biol Chem.

[36] Shen Q, Christakos S (2005). The vitamin D receptor, Runx2, and the notch signaling pathway cooperate in the transcriptional regulation of osteopontin. J Biol Chem.

[37] Sato M, Morii E, Komori T, Kawahata H, Sugimoto M, Terai K (1998). Transcriptional regulation of osteopontin gene in vivo by PEBP2alphaA/CBFA1 and ETS1 in the skeletal tissues. Oncogene.

[38] Bejarano L, Schuhmacher AJ, Méndez M, Megías D, Blanco-Aparicio C, Martínez S (2017). Inhibition of TRF1 Telomere Protein Impairs Tumor Initiation and Progression in Glioblastoma Mouse Models and Patient-Derived Xenografts. Cancer Cell.

[39] Huang D, Lu W, Zou S, Wang H, Jiang Y, Zhang X (2017). Rho GDP-dissociation inhibitor α is a potential prognostic biomarker and controls telomere regulation in colorectal cancer. Cancer Sci.

[40] Gialeli C, Nikitovic D, Kletsas D, Theocharis AD, Tzanakakis GN, Karamanos NK (2014). PDGF/PDGFR signaling and targeting in cancer growth and progression: focus on tumor microenvironment and cancer-associated fibroblasts. Curr Pharm Des.

[41] Farooqi AA, Siddik ZH (2015). Platelet-derived growth factor (PDGF) signalling in cancer: rapidly emerging signalling landscape. Cell Biochem Funct.

[42] Bolden Jessica E., Peart Melissa J., Johnstone Ricky W. (2006). Anticancer activities of histone deacetylase inhibitors. Nature Reviews Drug Discovery.

[43] Suraweera A, O'Byrne KJ, Richard DJ (2018). Combination therapy with histone deacetylase inhibitors (HDACi) for the treatment of Cancer: achieving the full therapeutic potential of HDACi. Front Oncol.

[44] Gray SG, Qian CN, Furge K, Guo X, Teh BT (2004). Microarray profiling of the effects of histone deacetylase inhibitors on gene expression in cancer cell lines. Int J Oncol.

[45] Reid G, Métivier R, Lin CY, Denger S, Ibberson D, Ivacevic T (2005). Multiple mechanisms induce transcriptional silencing of a subset of genes, including oestrogen receptor alpha, in response to deacetylase inhibition by valproic acid and trichostatin a. Oncogene.

[46] Wang Z, Zang C, Cui K, Schones DE, Barski A, Peng W (2009). Genome-wide mapping of HATs and HDACs reveals distinct functions in active and inactive genes. Cell.

[47] Liang T, Fang H (2018). Structure, functions and selective inhibitors of HDAC6. Curr Top Med Chem.

[48] Tao H, Chen YY, Sun ZW, Chen HL, Chen M (2018). Silence of HDAC6 suppressed esophageal squamous cell carcinoma proliferation and migration by disrupting chaperone function of HSP90. J Cell Biochem.

[49] Bertos NR, Gilquin B, Chan GK, Yen TJ, Khochbin S, Yang XJ (2004). Role of the tetradecapeptide repeat domain of human histone deacetylase 6 in cytoplasmic retention. J Biol Chem.

[50] Verdel A, Curtet S, Brocard MP, Rousseaux S, Lemercier C, Yoshida M (2000). Active maintenance of mHDA2/mHDAC6 histone-deacetylase in the cytoplasm. Curr Biol.

[51] Hubbert C, Guardiola A, Shao R, Kawaguchi Y, Ito A, Nixon A (2002). HDAC6 is a microtubule-associated deacetylase. Nature.

[52] Matsuyama A, Shimazu T, Sumida Y, Saito A, Yoshimatsu Y, Seigneurin-Berny D (2002). In vivo destabilization of dynamic microtubules by HDAC6-mediated deacetylation. EMBO J.

[53] Zhang Y, Li N, Caron C, Matthias G, Hess D, Khochbin S (2003). HDAC-6 interacts with and deacetylates tubulin and microtubules in vivo. EMBO J.

[54] Verdin E, Dequiedt F, Kasler HG (2003). Class II histone deacetylases: versatile regulators. Trends Genet.

[55] Medler TR, Craig JM, Fiorillo AA, Feeney YB, Harrell JC, Clevenger CV (2016). HDAC6 deacetylates HMGN2 to regulate Stat5a activity and breast Cancer growth. Mol Cancer Res.

