# Supplementary figures and images for: HDACs control RUNX2 expression in cancer cells through redundant and cell context-dependent mechanisms
Source: J Exp Clin Cancer Res. 2019 Aug 8;38:346. doi: 10.1186/s13046-019-1350-5 (PMC6686443; doi:10.1186/s13046-019-1350-5)

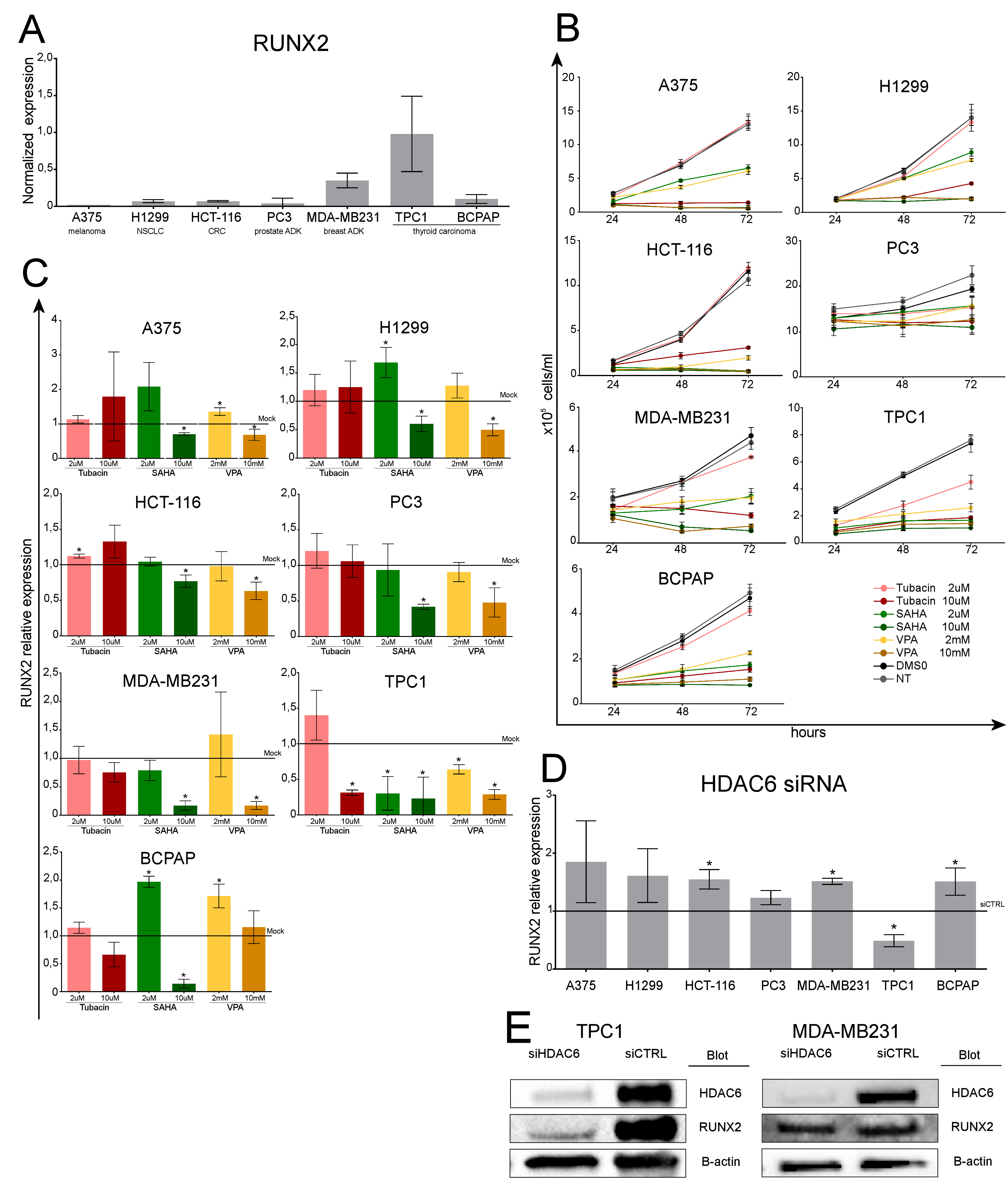

Supplement: Supplementary file 1 — Figure S1. a) Levels of HDAC6 mRNA in all cell lines 48 h after transfection with specific siRNA against HDAC6. b) RUNX2 expression levels 48 h after transfection with siRNA specific for HDAC1, HDAC2, HDAC3 and HDAC8 or with the combination of the former three, in BCPAP cells. c) expression levels of HDAC1, HDAC2, HDAC3 and HDAC8 48 h after transfection with the respective siRNA in TPC1, MDA-MB231 and BCPAP cells. d) expression levels of HDAC1, HDAC2 and HDAC3 48 h after transfection with a combination of the respective siRNAs in TPC1, MDA-MB231 and BCPAP cells. e) Representative ChIP experiments showing the binding of HDAC1 on the RUNX2 regulatory elements in BCPAP cells. f) Representative western blot showing basal levels of RUNX2, HDAC1 and HDAC6 in TPC1 and MDA-MB231 cells. C-Jun and ɑ-Tubulin were used as a control of correct nucleus-cytoplasm fractionation, this image is representative of all the fractionation performed for the co-IP experiments showed in Fig. 4g-h) Representative co-IP of HDAC1 with HDAC1 and c-Jun in TPC1 and MDA-MB231. i) Correlation of RUNX2 and HDAC6 expression in thyroid cancer samples from TCGA database. j) Representative western blot showing down-regulation of RUNX2 proteins 48 h after TPC1 cell transfection with specific siRNA. k) Basal expression levels of HDAC6 evaluated by qRT-PCR in all the tested cell lines. l) Correlation between the expression of RUNX2 and HDAC6. Where no otherwise specified, histograms represent the relative fold change +/− SD of silenced cells compared to control cells. Each experiment represents the average of at least two independent replicates. * p < 0.05 (TIF 3014 kb) [file 13046_2019_1350_MOESM1_ESM.tif]
