# Supplementary material for: HDACs control RUNX2 expression in cancer cells through redundant and cell context-dependent mechanisms
Source: J Exp Clin Cancer Res. 2019 Aug 8;38:346. doi: 10.1186/s13046-019-1350-5 (PMC6686443; doi:10.1186/s13046-019-1350-5)
Supplement: Supplementary file 2 — List of Interfering oligos, Primers and Antobodies. (DOCX 28 kb) [file 13046_2019_1350_MOESM2_ESM.docx]

| **Interfering oligos** | | | |
| --- | --- | --- | --- |
| *Gene target* | *Manufacturer* | *Code* | *Sequence / siRNA ID* |
| HDAC1 | Silencer Select RNAi (Thermo Scientific) | 4390824 | s73 |
| HDAC2 | Silencer Select RNAi (Thermo Scientific) | 4390824 | s6495 |
| HDAC3 | Silencer Select RNAi (Thermo Scientific) | 4390824 | s16876 |
| HDAC8 | Silencer Validated RNAi (Thermo Scientific) | AM51331 | 120597 |
| HDAC6 | TriFECTa DsiRNA Duplex (Integrated DNA Technologies) | custom sequences | Sense: rGrGrUrUrCrArCrArGrCrCrUrArGrArArUrArUrArUrUrGAT Anti-sense:rArUrCrArArUrArUrArUrUrCrUrArGrGrCrUrGrUrGrArArCrCrArA |
|  |  |  | Sense: rGrGrArGrArGrGrArGrArArCrCrUrArCrUrArGrGrArGrAGG Anti-sense:rCrCrUrCrUrCrCrUrArGrUrArGrGrUrUrCrUrCrCrUrCrUrCrCrUrG |
|  |  |  | Sense: rArGrArGrArArCrUrGrCrGrArCrGrArUrUrArArUrUrGrUGG Anti-sense: rCrCrArCrArArUrUrArArUrCrGrUrCrGrCrArGrUrUrCrUrCrUrUrA |
| RUNX2 | Stealth RNAi (Thermo Scientific) | 1299001 | HSS101401 |
|  |  |  | HSS101403 |
|  |  |  | HSS189448 |

Additionale file 2: Table S1

Additionale file 2: Table S2

| **Gene expression primers** | |  | **ChIP primers** | |
| --- | --- | --- | --- | --- |
| *Name* | *Sequence* |  | *Name* | *Sequence* |
| RUNX2 Fw | GCTCTTCTTACTGAGAGTGGAAGG |  | Neg Ctrl_Fw | TCTCAAGGTGCCTGTCTGC |
| RUNX2 Rv | GTGCCTAGGCGCATTTCA |  | Neg Ctrl_Rv | TGAAGTTTGGCCTCTGGTCT |
| HDAC1 Fw | CTCACCGAATCCGCATGACT |  | RUNX2 prom P2_Fw | ACCATGGTGGAGATCATCG |
| HDAC1 Rv | TCGCTGTGGTACTTGGTCAT |  | RUNX2 prom P2_Rv | GGCAGGGTCTTGTTGCAG |
| HDAC2 Fw | TGAAGCCTCATAGAATCCGCA |  | ENH3_Fw | GCTGGGAAGATAGCCAAGAA |
| HDAC2 Rv | TGTCATTTCTTCGGCAGTGG |  | ENH3_Rv | CCTTGCATCAGTTCCACAGA |
| HDAC3 Fw | TGTGATCGATTGGGCTGCTT |  | ENH11_Fw | CCCAAACCCCAAAGCAGAGA |
| HDAC3 Rv | TATAACCACCACCACCCAGC |  | ENH11_Rv | CCCAAGTTCTCACCAGGCAT |
| HDAC6 Fw | CGGTTTGCTGAAAAGGAAGA |  | ENH13_Fw | GTGGAGTGGAGAGAGGAGAA |
| HDAC6 Rv | TGTCTGCTAGGACACGGAGTT |  | ENH13_Rv | TGGCTTCATCTCACCCTCAG |
| HDAC8 Fw | GTCGCTGGTCCCGGTTTATA |  | SPP1 prom_Fw | CATAGCGGGTCATTGTTGGG |
| HDAC8 Rv | GCTTCAATCAAAGAATGCACCA |  | SPP1 prom_Rv | CAGCGGGATAGAACACTCCT |
| GUSB Fw | TTGAGCAAGACTGATACCACCTG |  | TERF1 prom_Fw | CAATCAGAAGCCGGAACAGG |
| GUSB Rv | TCTGGTCTGCCGTGAACAGT |  | TERF1 prom_Rv | TGTTAAATGGCTCGCTTGGG |
| SPOCD1 Fw | CTACGACCTGGTGCGGATG |  | PDGFB prom_Fw | AGGAGAAGTTGCCACCCTTT |
| SPOCD1 Rv | GCTCCTTCTGTTGCTGCTCA |  | PDGFB prom_Rv | AGAGGAAAGGCTGTCTCCAC |
| MMP1 Fw | CATGACTTTCCTGGAATTGG |  |  |  |
| MMP1 Rv | CCTGCAGTTGAACCAGCTAT |  |  |  |
| PUMA Fw | GACCTCAACGCACAGTACGA |  |  |  |
| PUMA Rv | CTGGGTAAGGGCAGGAGTC |  |  |  |
| RHOB Fw | TCGAGAACTATGTGGCCGAC |  |  |  |
| RHOB Rv | CTGTCCACCGAGAAGCACAT |  |  |  |
| EFR3B Fw | GCTGCTAAAATCCCTCCTGC |  |  |  |
| EFR3B Rv | CGGTCCAGGAAGTTGCTAGG |  |  |  |
| FAM83A Fw | TTCGTTTGTGTGCTCCTGGA |  |  |  |
| FAM83A Rv | CTCTCCTTCCACACTCCGGA |  |  |  |
| HAS2 Fw | CATGGTTGGAGGTGTTGGGG |  |  |  |
| HAS2 Rv | CATGGTTGGAGGTGTTGGGG |  |  |  |
| TINAGL1 Fw | GCCCATCTTCCTCGGTCATG |  |  |  |
| TINAGL1 Rv | GCTCATGAATCAGGTTGGGC |  |  |  |
| NUCKS1 Fw | AGAGGAGGATGAGGCACCAT |  |  |  |
| NUCKS1 Rv | GGAAATCTTCATCGCTGCCG |  |  |  |
| STOX1 Fw | GCAGGAATCACTTTTGGAGCG |  |  |  |
| STOX1 Rv | TAATCAGCGTTCCCAGAGTG |  |  |  |
| SCD Fw | TGCAAGTTCTACACCTGGCT |  |  |  |
| SCD Rv | CTCCACAGACGATGAGCTCC |  |  |  |
| ADD3 Fw | TGGAGCAGAGGAAACGAGTT |  |  |  |
| ADD3 Rv | GGCATTCCAAGTCTTCCCGA |  |  |  |
| SYT12 Fw | CCATGATCTTCTCGGTGCCA |  |  |  |
| SYT12 Rv | CTGACGGCCCAATGATGACA |  |  |  |
| CTD-2531D15.4 Fw | ACAATACTCATCCAGGCGGA |  |  |  |
| CTD-2531D15.4 Rv | TGAGGCTGCCATGGAACTTT |  |  |  |
| LGR5 Fw | AGTCAGCTGCTCCCGAATC |  |  |  |
| LGR5 Rv | CAGTGAATGCTCCCTTGGGA |  |  |  |
| PLCG2 Fw | ATGGCACTCAGTTCGTCCTC |  |  |  |
| PLCG2 Rv | GTGGACGCATTCATCGCTTC |  |  |  |
| PIK3R6 Fw | TGTCACAGACCACACATGTC |  |  |  |
| PIK3R6 Rv | TGGATCTGGATATTGTTCGTCC |  |  |  |
| TMEM163 Fw | ATCCTGGCCGTGTTGAAGTT |  |  |  |
| TMEM163 Rv | CGCGCTCAGAAGAATGGAGA |  |  |  |
| SCN9A Fw | TGATGGTCATGGTCATTGGAA |  |  |  |
| SCN9A Rv | TGAGCTCAATAATAAGGCCAGAA |  |  |  |
| IDH1 Fw | AGACAATTGAGGCTGGCTTCA |  |  |  |
| IDH1 Rv | CAAGTAGTCAGAACGTTGCACA |  |  |  |
| PDGFB Fw | CACTCGATCCGCTCCTTTGA |  |  |  |
| PDGFB Rv | CGGGTCATGTTCAGGTCCAA |  |  |  |
| SLC6A6 Fw | GCTTCCCGTACCTCTGCTAC |  |  |  |
| SLC6A6 Rv | AAATACGGTATGAGAAACGCAC |  |  |  |
| CHRD Fw | GGGAAGGAGAGTCGATGCTG |  |  |  |
| CHRD Rv | CCTCTGGCTGCTCCCTAAGA |  |  |  |
| SPP1 Fw | GACTCGTCTCAGGCCAGTTG |  |  |  |
| SPP1 Rv | AGGAGGCAAAAGCAAATCACTG |  |  |  |
| SLCO4C1 Fw | TGCTTCACTACTGCCTCTTGG |  |  |  |
| SLCO4C1 Rv | ACGCTTCTCAACAGTGGAAA |  |  |  |
| FAM20C Fw | CAAAGCGGCGGAGAACCC |  |  |  |
| FAM20C Rv | GATAGGAGTCCACGGCCG |  |  |  |
| TERF1 Fw | GTTTCAAAGAGTCAGCCGGT |  |  |  |
| TERF1 Rv | TCCTCACGCCAGATCTCAAA |  |  |  |
| GYG2 Fw | GCCTTCTCTCCACACGCATA |  |  |  |
| GYG2 Rv | TCAGTAAGCCTTGGTCTGCC |  |  |  |
| HPRT Fw | AGACTTTGCTTTCCTTGGTCAGG |  |  |  |
| HPRT Rv | GTCTGGCTTATATCCAACACTTCG |  |  |  |
| GAPDH Fw | ATTGGGCGCCTGGTCAC |  |  |  |
| GAPDH Rv | AACATGTAAACCATGTAGTTGAGGTCA |  |  |  |
| RPS7 Fw | TTCTGCCTAAGCCAACTCGAAAA |  |  |  |
| RPS7 Rv | CGGATTCTCTTGCCCACAATTTC |  |  |  |

Additionale file 2: Table S3

| **Antobodies** | | | | | |
| --- | --- | --- | --- | --- | --- |
|  |  |  | *Experimental conditions* | | |
| *Name* | *Code* | *Manufacturer* | ChIP | Co-IP | Western Blot |
| Anti-HDAC1 | 17-10199 | Millipore | 1ug for >20*10^6^ cells | 2ug for >30*10^6^ cells | 1:1000 |
| Anti-HDAC6 | BK7558S | Cell Signaling | 5ug for >20*10^6^ cells | 1ug for >40*10^6^ cells | 1:1000 |
| Anti-H3K27Ac | ab4729 | Abcam | 1ug for >20*10^6^ cells | // | // |
| Anti-RUNX2 | AF2006 | R&D systems | 7ug for >20*10^6^ cells | 10ug for >40*10^6^ cells | 1:1000 |
| Anti-c-Jun | 60A8 | Cell Signaling | // | // | 1:1000 |
| Anti-αTubulin | sc-8035 | Santa Cruz | // | // | 1:1000 |
| Anti-Yap | 14074S | Cell Signaling | 1ug for >20*10^6^ cells | // | 1:1000 |
| Anti-Rpb1 NTD (D8L4Y) | 14958 | Cell Signaling | 5ug for >20*10^6^ cells | // | 1:1000 |
| Anti-Beta actina | A1978 | Sigma - Aldrich | // | // | 1:2000 |
| Normal Rabbit IgG | 2729S | Cell Signaling | corresponding to each specific antibody | | // |
| Normal Goat IgG | sc-2028 | Santa Cruz | corresponding to each specific antibody | | // |
| Anti-rabbit IgG-HRP | NA934V | GE healthcare | // | // | 1:5000 |
| Anti-mouse IgG-HRP | NXA931V | GE healthcare | // | // | 1:5000 |
| Anti-goat IgG-HRP | sc-2350 | Santa Cruz | // | // | 1:5000 |
